# Comparative Studies on Detergent-Assisted Apocytochrome b_6_ Reconstitution into Liposomal Bilayers Monitored by Zetasizer Instruments

**DOI:** 10.1371/journal.pone.0111341

**Published:** 2014-11-25

**Authors:** Michał A. Surma, Andrzej Szczepaniak, Jarosław Króliczewski

**Affiliations:** Faculty of Biotechnology, University of Wroclaw, Wroclaw, Poland; The University of Tennessee Health Science Center, United States of America

## Abstract

The present paper is a systematic, comparative study on the reconstitution of an apocytochrome b_6_ purified from a heterologous system using a detergent-free method and reconstitution into liposomes performed using three different detergents: SDS, Triton X-100 and DM, and two methods of detergent removal by dialysis and using Bio-Beads. The product size, its distribution and zeta potential, and other parameters were monitored throughout the process. We found that zeta potential of proteoliposomes is correlated with reconstitution efficiency and, as such, can serve as a quick and convenient quality control for reconstitution experiments. We also advocate using detergent-free protein purification methods as they allow for an unfettered choice of detergent for reconstitution, which is the most crucial factor influencing the final product parameters.

## Introduction

Membrane proteins account for 20–25% of all open reading frames in sequenced genomes, and fulfill a wide range of functions in cells [1]. Our knowledge of the assembly of this class of proteins is still poor. The study of membrane proteins *in situ* is difficult for several reasons: proteins assemble in larger complexes; interact with other proteins, and with other cell components. Therefore model bilayer *in vitro* systems are often used for studying membrane proteins as they negate many of the problems associated with the use of membrane vesicles [2]. Performing *in vitro* studies requires solubilization of a membrane protein with a suitable detergent, followed by a functional reconstitution in a well-characterized bilayer system. However such studies are greatly hampered due to the difficulties in purifying membrane proteins in large amounts, which is the first limitation in this approach.

Two types of reconstitution system are routinely used: planar lipid bilayers and liposomes. The planar lipid bilayer system consists of a phospholipid bilayer supported within an approximately 1 mm diameter pore in a hydrophobic partition separating two aqueous chambers [3]. The liposome system consists of spherical lipid bilayers enclosing an aqueous compartment and separating it from the aqueous milieu [4]. Planar lipid bilayers have the advantage of a well-defined, though enforced, geometry and simple to assay transport between the aqueous compartments on either side of a membrane. In comparison to liposomes, the major disadvantages of planar bilayers include difficulties in controlling the extent of reconstitution of the protein into the bilayer and measuring the exact amount that has been reconstituted [5]. Planar lipid bilayers are also inherently unstable and very sensitive to the presence of free detergent [6]. Moreover, liposomal lipid composition is easy to manipulate, which taken together with the ease of controlling reconstituted protein amounts made proteoliposomes the leading tool for characterizing transmembrane transport by different membrane proteins [7]–[10].

Liposomes can be described by a number of properties. Two of the most important are particle size and zeta potential. Almost all particulate or macroscopic materials in contact with a liquid acquire an electronic charge on their surfaces. Zeta potential is an indicator of this charge that can be used to predict and control the stability of colloidal suspensions or emulsions and also is important for interaction of liposomes with biological systems *in vivo* [11].

In the present work, we have applied different detergents and methods of their removal, followed by measurements of zeta potential and other properties to investigate the process of reconstitution of apocytochrome b_6_ into liposomes. Cytochrome b_6_ is present in chloroplasts and functions in the b_6_f complex that is localized between green plants photosystems I and II. It is an integral membrane protein with a mass of about 24 kDa that contains three hemes as cofactors. It is the most hydrophobic part of the b_6_f complex and is comprised of four transmembrane helices [12]. Like many other integral proteins, cytochrome b_6_ operates with an unknown uncleaved signal for membrane insertion and integration [13]. Previously it was shown that apocytochrome b_6_ from spinach can be heterologously expressed as a fusion protein – maltose binding protein-apocytochrome b_6_ (MBP-b_6_) in *E. coli* – and that the expressed fusion protein integrates into the *E. coli* inner membrane [14]–[16]. In other reports it was reported that transmembrane cytochrome b_6_ assembles spontaneously *in vitro* in the *E. coli* membrane [17]. The maltose binding protein is part of the maltodextrin transport system in E. coli that belongs to the periplasmic permease family. It is synthesized as a precursor in the cytoplasm and must be exported to the periplasm where it is folded and becomes functional. [18]. MBP serves as an initial high-affinity binding component of the active-transport system of maltooligosaccharides in bacteria and also participates in chemotaxis towards maltooligosaccharides. It is a monomeric protein with a mass of about 40 kDa and contains two globular domains separated by a deep groove with the oligosaccharide-binding site. Both domains exhibit similar packing of the secondary structure elements; they are composed of a core of β sheet flanked on both sides by helices. The maltose-binding protein consists of 40% α helix and 30% β sheet [19], [20].

## Materials and Methods

Egg phosphatidyl choline (EPC) and cholesterol were purchased from Lipid Products (England). Triton X-100, *n*-dodecyl-β-D-maltoside (DM) and sodium dodecyl sulfate (SDS) detergents were obtained from Sigma-Aldrich (USA). SM2 Bio-Beads (20–50 mesh; Bio-Rad) were prepared for application by extensive rinsing with methanol and 20 mM HEPES/HCl, pH 7.4 buffer [21]. 100 nm polycarbonate filters were purchased from Nucleopore Corporation. All other reagents were of analytical grade. MBP-apocytochrome b_6_ fusion protein (MBP-b_6_) was isolated using the *E. coli* overexpression system according to Króliczewski and Szczepaniak [14]. MBP-b_6_ in detergent solutions (Triton X-100, DM or SDS) was prepared by exchange dialysis to 20 mM HEPES/HCl, pH 7.4 buffer containing respectively 0.1% Triton X-100, 0.025% DM or 0.3% SDS.

### Circular dichroism (CD) measurements

Protein renaturation in detergent solutions was characterized by obtaining circular dichroism spectra recorded at RT in a quartz cuvette using a cell with a 1-mm light path from 190 to 260 nm, averaging 10 scans per sample with 1 nm step, with Jasco J-715 apparatus (JASCO Europe S.R.L, Italy). The spectra were noise reduced using the instrument algorithm and corrected for the buffer contributions without peak shape distortion and without altering peak maxima or intensities. Secondary structure content was estimated using the CDPro software (http://lamar.colostate.edu/~sreeram/CDPro), which permits analysis of a secondary structure by CONTIN, SELCON, and CDSSTR [22], [23].

### Liposome preparation

7∶3 molar mixture of chloroform solutions of EPC and cholesterol was dried in a low-pressure rotary evaporator to obtain a thin film of lipids on the wall of round-bottomed flask. Multilamellar vesicles were prepared by rehydrating the dry lipid film with 20 mM HEPES/HCl, pH 7.4 buffer; the rehydration was assisted by agitation with glass beads. Unilamellar liposomes of defined size were prepared by ten times extrusion of multilamellar vesicles through 100 nm Nucleopore filters using LIPEX extruder (Lipex Biomembranes, Canada) [24], [25].

### Liposome solubilization

Solubilization of liposomes was carried out by adding an increasing amount (1 to 200 µL) of a concentrated stock of detergents (the same volume of buffer was used as a control) to 1 mL liposome suspensions with a lipid concentration of 2.5 mg/mL with constant stirring at 20°C and monitored by the light scattering at a 90° angle (nephelometry) at 600 nm with Kontron SFM 25 (Kontron AG, Switzerland) spectrofluorimeter in 1 cm × 1 cm acrylic cuvettes (Sarstedt, Germany). The onset solubilization detergent concentration value was set as an end point of a stage where the light scattering shows a decrease followed by a slight increase (the partitioning of detergent monomers between the aqueous medium and the lipid bilayer); and the total solubilization point was set at the inflection point after which a large and significant decrease in scattered light (gradual liposome solubilization and lipid-detergent micelles forming) slowed down to a stage where the addition of detergent stopped decreasing the light scattering rapidly (e.g. the complete solubilization of lipid into small mixed micelles) [26].

### Size distribution and zeta-potential of liposomes

The size and size distribution [27] and zeta-potential [28] of vesicles were measured using a Malvern Zetasizer 2000 instrument (Malvern Instruments, Malvern, UK). Samples were diluted to a total lipid concentration of approximately 0.25 mg/ml in 20 mM HEPES/HCl, pH 7.4 prior to measurements. Zetasizer measures distribution of size in a population and the sizes provided in Tables 1–3 reflect peaks of this distribution in nm. Each peak is characterized by a width at its base (in nm), which reflects homogeneity (or polydispersity) of the subpopulation under this peak. Each sample was measured five times and the result is expressed as an average value ± standard deviation.

**Table 1:** Reconstitution of protein using SDS.

	Diameter (peaks [nm])		Homogeneity (width of a peak [nm])	
	Control	Reconst.	Control	Reconst.
**Dialysis**				
LUV	113.7±11.2		69.9±15.0	
Post. mix.	94.4±8.8	93.5±8.6	56.4±13.6	55.3±9.6
Proteolip.	107.5±14.5	106.0±10.7	37.2±7.8	32.9±7.5
**Bio-Beads**				
LUV	116.9±10,2		75.4±14.8	
Post. mix.	85.3±9.3	83.3±9.4	54.1±14.6	57.2±18.8
Proteolip.	101.6±12.5	96.9±8.4	59.4±14.2	59.4±11.0

The size and the size distribution of reconstituted vesicles.

LUV – initial liposome solutions (the same initial batch was measured at this point, which later was divided in two and processed in parallel for control and reconstitution experiments); Post. mix. – proteoliposomes in post-reconstitution mixture (directly after detergent removal); Proteolip. – final proteoliposomes (after centrifugation and washing). Control experiments underwent the same routine only the detergent solution without protein was added to them. Given are the mean values of n = 5 independent experiments with standard deviations.

**Table 2:** Reconstitution of protein using Triton X-100 removed with Bio-Beads.

	Diameter (peaks [nm])		Homogeneity (width of a peak [nm])	
	Control	Reconst.	Control	Reconst.
LUV	126.2±14,0		75.2±20.3	
Post. mix.	96.2±10.9	97.8±8.1	49.3±10.5	54±8.9
	386.6±55.6	494.3±78.5	165.4±45.8	377.1±98.0
Proteolip.	101.7±15.4	108.9±15.2	50.3±10.2	56.2±14.0
	400.3±89.5	498.7±57.4	167.5±28.5	172.2±39.0

The size and the size distribution of reconstituted vesicles.

Description the same as for the Table 1.

**Table 3:** Reconstitution of protein using DM.

	Diameter (peaks [nm])		Homogeneity (width of a peak [nm])	
	Control	Reconst.	Control	Reconst.
**Dialysis**				
LUV	122.5±15.2		65.6±18.9	
Post. mix.	198.2±20.1	208.5±19.8	72.7±15.1	102.7±22.8
	421.0±68.3	454.7±69.3	73.3±12.2	130.7±26.0
	755.7±105.3	828.4±105.6	110.4±26.5	146.5±35.5
	1200.8±110.8		146.8±35.0	
Proteolip.	163.6±17.2	443.4±58.0	82.1±19.9	89.1±19.9
	652.8±98.7	829.4±79.6	551.4±155.3	571±128.8
**Bio-Beads**				
LUV	108.1±14,5		62.6±10.3	
Post. mix.	138.8±18.0	140.3±16.7	93.1±18.4	85.9±17.4
Proteolip.	146.9±16.0	149.4±19.5	104.1±25.0	95.2±14.4

The size and the size distribution of reconstituted vesicles.

Description the same as for the Table 1.

### MBP-b_6_ protein reconstitution

Liposomes with a lipid concentration of 2.5 mg/mL in 1 mL of total volume were first treated with detergents (105 µL of of DM and Triton X-100 and 2 µL a concentrated stock solution of SDS was added) to reach the total solubilization concentration indicated by a decrease and subsequent stabilization of light scattering. Then, detergent solution of protein (15 µL of 800 µg/mL solution) was added to the equilibrated detergent-lipid mixtures to obtain 1∶200 protein to lipid weight ratio. Finally, proteoliposomes were reconstituted by two different methods of detergent removal:

A. Detergent removal by dialysis. Detergent-lipid-protein mixtures were transferred to Roth QuickStep Micro Dialyzer System and dialysed at 4°C through 30 kDa Sigma-Aldrich dialysis membrane toward 20 mM HEPES/HCl, pH 7.4 buffer, four times for 1 h to 100 volumes and overnight to 150 volumes of buffer [29].

B. Detergent removal by Bio-Beads [21], [26], [30]. 300 mg/mL of wet Bio-Beads was added to equilibrated detergent-lipid-protein mixtures and everything was incubated for 6 h at 20°C with constant gentle stirring.

The resulting proteoliposomes was recovered from the ∼1 mL volume of post-reconstitution mixture by centrifugation for 30 min at 65000 x g [31]–[33], washed three times and finally resuspended in 1 mL of 20 mM HEPES/HCl, pH 7.4 buffer.

### Efficiency of the MBP-b_6_ protein reconstitution

To calculate the efficiency of the protein reconstitution, the protein concentration in proteoliposomes was measured using Bicinchoninic Acid Kit Sigma-Aldrich (St Louis, MO, USA) according to the producer protocol with control samples (no protein reconstituted) as blanks. Each sample was measured three times and the result is expressed as an average value ± standard deviation.

### Determination of protein orientation in bilayer

To the proteoliposome fraction suspended in 100 µl of 20 mM HEPES/HCl, pH 7.4 buffer with 7.5 mM CaCl_2_, proteinase K was added to a final concentration of 0.33 mg/mL and the mixture was incubated at 30°C for 1 h. After incubation time PMSF inhibitor was added to a final concentration of 0.66 mg/mL to stop proteolytic activity. Proteoliposomes were pelleted by ultracentrifugation as described above and the supernatant was discarded. Pellets were dissolved in 100 µL of 10% SDS and 33 µL of 36% trichloroacetic acid was added and mixture was incubated for 1 h on ice in order to precipitate protein. After centrifugation at 16000 x g the supernatant was discarded and the pellet was washed once with cold acetone. Samples were air-dried and prepared for SDS-PAGE. Densitometric analysis was performed using the Fiji distribution (http://fiji.sc) of ImageJ (http://http://imagej.nih.gov/ij) software ver. 1.49b and Java 1.6.0_65, using 600 dpi, 32-bit deep, black and white scanned image.

### Western blot analysis

SDS-PAGE was performed using standard techniques and was followed by a semidry blotting. Antibodies raised against MBP were prepared in rabbits [34]. The protein was visualized using secondary peroxidase-conjugated goat anti-rabbit antibodies (Sigma-Aldrich, St Louis, MO, USA).

### Statistical analysis

The two-tailed, unpaired t-test using the Holm-Sidak method, with alpha = 0.05 was used for determining the statistical significance of differences between the means for reconstituted and control, using Prism 6.0e (GraphPad Software Inc., La Jolla, CA, USA).

## Results

### Secondary protein structure in detergent solutions

**Figure 1:**
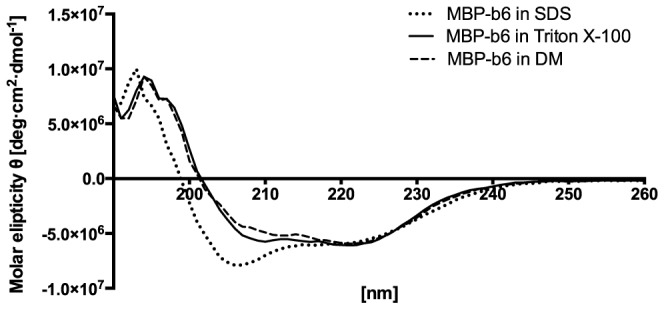
Circular dichroism spectra of MBP-cytochrome b_6_ fusion protein in different detergent solutions.

To test whether MBP-b_6_, isolated from overexpression bacterial system, maintains its native helical-rich structure after solubilization in a given detergent, circular dichroism spectrometry in the ultraviolet wavelength region (UV-CD) was performed followed by secondary structure analysis to estimate structure content and thus the protein folding state. Secondary structure analysis (Table 4) shows that the protein in DM, as well as in Triton X-100 solution, recovered its structure. However the spectra obtained from SDS solution shows decreased ellipticity in the range between 200–210 nm, which indicates incomplete secondary structure recovery (Figure 1 and Table 4).

**Table 4:** Predicted per cent secondary structure content of the MBP-b_6_ in detergent solutions (Triton X-100, DM or SDS) from CD analysis.

	Secondary structure type			
	Helix	Sheet	Turn	Unordered
Calculated from the crystal structure of cytochrome b_6_ (PDB code 1Q90) and MBP (PDB code 1PEB)	54,7	20,7	3,8	20,8
MBP-b_6_ in DM	44,7	19,1	3,1	33,1
MBP-b_6_ in Triton X-100	41,9	18,7	3,4	36,0
MBP-b_6_ in in SDS	27,3	25,4	5,1	42,2

The percent secondary structure of the CD spectra was predicted using the CDSSTR algorithm found in the CDPro software suite.

### Solubilization of liposomes by DM, Triton X-100 and SDS

In order to reconstitute our protein from an isotropic solution of lipid-protein-detergent and lipid-detergent micelles, we first had to determine the detergent concentration that was necessary to fully solubilize the liposome suspension [35]. Previous studies have demonstrated that measuring changes in the optical properties of liposome suspensions upon treatment with detergents can serve as a convenient technique for tracing bilayer solubilization [35]. The solubilization perturbations occurring by gradual addition of detergents to liposomes, yielding a suspension turbidity decrease, which results in lower light scattering, were measured in nephelometrical manner.

**Figure 2:**
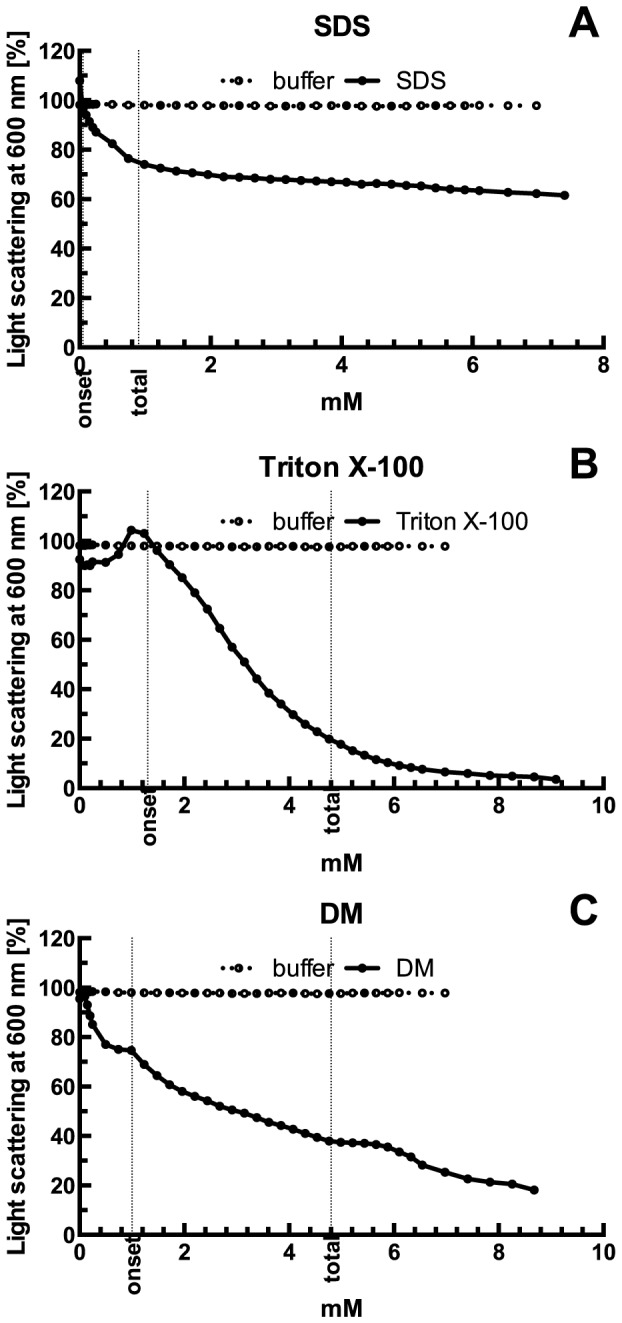
Solubilization of liposomes (black circles) by SDS (A), Triton X-100 (B) and DM (C) monitored by light scattering. The onset of solubilization concentration and the total lysis concentration are marked with dashed lines. As a control, a buffer without detergent (empty circles) was titrated into liposomes.

The solubilization curves of 1 mL liposomal suspensions with a lipid concentration of 2.5 mg/ml upon adding increasing amounts of the detergents used in this study were plotted as described previously [26]. The onset and total solubilization detergent concentration values were determined for DM ≈1.0 and ≈4.8 mM, Triton X-100 ≈1.3, ≈4.8 and SDS ≈0.05 and ≈0.9 mM respectively (Figure 2A–C). DM and Triton X-100 5 mM and SDS 1 mM concentrations were used in reconstitution experiments as completely solubilizing ones. However, because detergents were added externally, the time needed for full equilibration of the solution had to be determined. For the above lipid concentrations, experiments indicated that around 30 min to 1 h was needed to get a constant value of light scattering.

### Liposomes and proteoliposome reconstitution

The overexpression system combined with a detergent independent method of protein purification allowed us to test the application of three different detergents in proteoliposome reconstitution. To follow the process of reconstitution, several parameters were measured during the procedure at several “checkpoints”.

**Figure 3:**
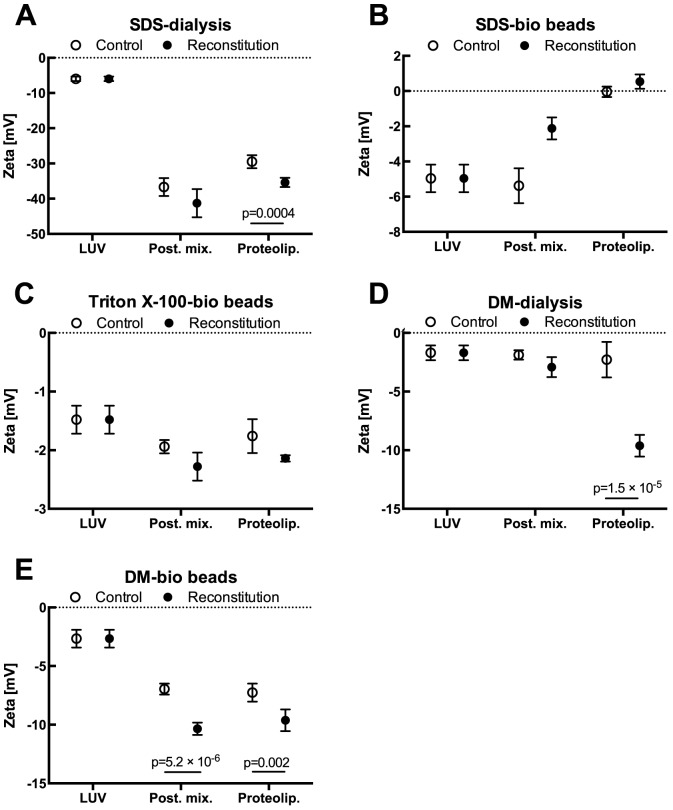
Zeta potential of liposomes during the reconstitution process for SDS removed by dialysis (A) or with Bio-Beads (B); Triton X-100 removed with Bio-Beads (C) and DM removed by dialysis (D) or with Bio-Beads (E). Plotted are the mean values of n = 5 independent experiments with error bars depicting standard deviations. Asterisks denote significant differences between control and reconstitution experiments. LUV – initial liposome solutions (the same initial batch was measured at this point, which later was divided in two and processed in parallel for control and reconstitution experiments); Post. mix. – proteoliposomes in post-reconstitution mixture (directly after detergent removal); Proteolip. – final proteoliposomes (after centrifugation and washing). Control experiments underwent the same routine only the detergent solution without protein was added to them.

The following procedure was used. Particles size and size distribution, zeta-potential values and light scattering were measured for initial liposome solutions (LUV on Figure 3 and Tables 1–3). Then a given detergent was added to reach the total solubilizing concentration and after a 1 h incubation light scattering was measured to ensure total solubilization. Next, the proper detergent solution of protein was added and once again light scattering was measured. With every detergent used, liposomes were shown to be solubilized completely and no further significant changes in light scattering were observed after protein addition. After an additional 1 h incubation to equilibrate the mixtures, detergent was removed using a chosen method. Proteoliposomes in the post-reconstitution mixture (Post. mix. on Figure 3 and Tables 1–3) were then characterized by size and size distribution and zeta-potential was determined. Finally, to separate proteoliposomes from the remaining mixture, centrifugation was applied and again size and size distribution and zeta-potential measurements were carried out, as well as protein concentration for these final proteoliposomes (Proteolip. on Figure 3 and Tables 1–3). The difference in size and size distribution between the control experiment (liposomes reconstituted with the same detergent and by the same manner but in the absence of protein) and normal proteoliposomes were insignificant. This proves that reconstituted protein does not affect or only minimally affects these parameters, however significant changes in zeta-potential were observed.

### Reconstitution using SDS

In this study, we used SDS mainly as a negative control in reconstitution. Using dialysis to remove detergent from solution one is able to reform liposomes after SDS solubilization. The obtained vesicles are characterized by a relatively small size and a narrow size distribution (Table 1). However reconstitution efficiency, indicated by a protein concentration in proteoliposomes, is almost close to zero (1.1±0.9%; n = 3). Additionally, zeta-potential seems to be strongly influenced by the presence of this anionic detergent and after detergent dialysis it maintains highly negative values (Figure 3A; n = 5, p = 0.0004). Quite similar results were obtained for SDS removed with Bio-Beads (Table 1). Again we were able to reconstitute vesicles of uniform size, but protein reconstitution efficiency was virtually 0%. Here, zeta-potential values were not negative (Figure 3B). These two experiments confirmed that SDS should not be used for the reconstitution of MBP-apocytochrome b_6_ into liposomes and casting uncertainty for its application with other proteins.

### Reconstitution using Triton X-100

Reconstitution of proteoliposomes using this detergent was also performed using two methods of removal: using dialysis and adsorptive Bio-Beads. According to expectations, it was impossible to decrease Triton X-100 concentration to levels sufficient for proteoliposomes reconstitution even after four days of dialysis. On the other hand, Triton X-100 removal with Bio-Beads led to a vesicle population with heterogeneous size distribution with two main size peaks at around 100 nm and 500 nm, however, vesicles of near micrometer size were also counted (Table 2).

Protein reconstitution efficiency of that method reached 15.5±5.8% (n = 3). Also negative zeta-potential increased after protein incorporation in comparison to the control experiment, which led to the hypothesis that these changes are caused by the protein presence in bilayer (Figure 3C).

### Reconstitution using DM

After liposome solubilization in DM, detergent was removed either using Bio-Beads adsorptive material or during a dialysis procedure. In the latter case, the recreated vesicles had sizes between 400–800 nm and part of the population was even larger than 1000 nm, suggesting the presence of large, multilamellar structures (Table 3). In this case protein reconstitution efficiency reached 29.1±4.2% (n = 3). Together with high reconstitution efficiency, the higher negative zeta-potential for proteoliposomes, in comparison with the protein free control, was observed (Figure 3D; n = 5, p = 1.5×10-5). DM removal using Bio-Beads also led to proteoliposome formation. This method yielded a very uniform vesicle population; with an average size around 140 nm and a very narrow size distribution (Table 3). Reconstitution efficiency was very high and reached 46.5±5.9% (n = 3) of the MBP-b_6_ added for reconstitution. Zeta-potential decrease was observed for these proteoliposomes as well, and in comparison with control liposomes proved to be highly significant (Figure 3E; n = 5, p = 0.002).

**Figure 4:**
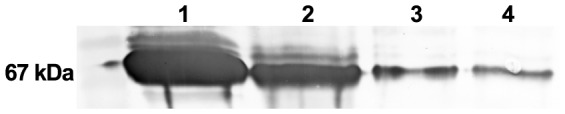
Orientation of MBP-apocytochrome b_6_ in proteoliposomes reconstituted with dodecyl maltoside removed with Bio-Beads was analyzed by Western blotting with anti-MBP antibodies. Purified, concentrated MBP-apocytochrome b_6_ in DM solution (1); DM solution of MBP-apocytochrome b_6_ added for reconstitution (2); untreated proteoliposomes (3); proteoliposomes treated with proteinase K (4). Lanes 3 and 4 contain the same amount of proteoliposomes.

For proteoliposomes produced from DM solutions and detergent removed by Bio-Beads the protein orientation in the bilayer was also checked. To this end, the proteoliposomal fraction was treated with proteinase K, in order to cleave off and digest outward-directed MPB domains of MBP-b_6_ fusion protein. The proteinase untreated fraction incubated under the same conditions served as a control. After incubation long enough to ensure total digestion of available MBP domains, SDS-PAGE and Western-blot with antibodies directed against MBP epitope (Figure 4) of obtained samples were performed and band intensities of proteinase treated and untreated samples were compared densitometrically. The ratio of outward-directed, MBP outside, protein (proteinase treated) to total protein incorporated in liposomes (untreated) was ∼53% (n = 1). This means almost equal ratio of inward and outward pointed protein, indicating symmetrically reconstituted proteoliposomes (1:1 N- and C-termini out *vs*. in).

## Discussion

Bilayer restoration upon detergent removal from homogenous solution of mixed lipid-detergent micelles has been proved to be an opposite process of bilayer solubilization [36] [37]. However the influence of reconstituted proteins on the properties of the end product (proteoliposomes) has not been examined in detail. In addition the choice of detergent is dictated mostly by the procedure used in protein purification [38], which often limits the application of different detergents in reconstitution experiments. In this work we compared the application of three different detergents: SDS, Triton X-100 and DM, removed by either dialysis or adsorption by Bio-Beads, in reconstituting apocytochrome b_6_ in liposomes.

When purifying proteins from natural membranes, the choice of detergent is dictated on the one hand by its ability to effectively solubilize membranes, allowing efficient purification, and on the other hand by its stabilizing properties and compatibility with downstream purification procedures. However, purification yield from natural membranes is usually not high, making it difficult to obtain sufficient quantitates for reconstitution experiments. On the contrary, heterologous overexpression systems ensure high yields and purification procedures that do not rely on detergents (e.g. gel filtration of inclusion bodies solubilized in urea or GdCl), but a resultant protein must be refolded to its native structure. Here various detergents can be used, as long as they ensure proper folding. We used protein purified from an overexpression system; therefore we tested detergents for protein refolding first.

Folding was induced by dialysis of the urea-solubilized MBP–apocytochrome b_6_ to a buffer containing an appropriate detergent. As shown above (Figure 1), the renaturation in Triton X-100 and DM was satisfactory. CD spectra and secondary structure analysis (Table 4) show that the protein recovered its native structure. As expected in SDS the decrease of ellipticity in the range of 200–210 nm was observed, indicating incomplete folding [14]. In previous studies it was shown that the disordered structure of the urea-solubilized heme-free form of cytochrome b_6_ in the presence of SDS was converted into a partially folded structure with 37% α-helical content [14]. Incomplete restoration of the native structure could be a crucial cause of the reduced ability to reconstitute a protein into the bilayer.

Due to its strong surfactant properties, SDS is not so mild in its effect as other, non-ionic detergents [37]. However it is frequently used as a membrane mimetic environment in NMR studies of transmembrane peptides and the involvement of electrostatic interactions in the mechanism of peptide folding induced by SDS binding has been described [39]. However, despite the fact that its high critical micelle concentration allows for its effective removal from solutions using dialysis, this detergent is not commonly used in reconstitution experiments. Thus, it follows that, from the perspective of reconstitution, in this study SDS proved to be completely useless. Although vesicle restoration was observed in both experiments with SDS removed by dialysis or Bio-Beads, the protein incorporation rate into the bilayer (reconstitution efficiency) was virtually zero. It is worth mentioning that after dialysis a significantly lower negative zeta-potential of reformed vesicles was observed for a protein reconstituted sample than for a control sample (Figures 3A and B). In general a very low zeta potential suggests that negatively charged SDS was not completely removed from bilayers in this process. And the decreased zeta-potential of the protein-reconstituted sample indicates that SDS remained bound to trace amounts of bilayer-incorporated protein (the reconstitution efficiency was exactly 1.1% in this case). It is a well-established fact that SDS interacts with proteins, binds to them and grants them a high negative charge in an almost stoichiometric manner, which is then the basis for the standard SDS-PAGE [40]. This conclusion is further supported by the result of our next experiment where SDS was removed with Bio-Beads, which yielded 0% reconstitution efficiency and where zeta potential did not remain negative. It also provided initial evidence that zeta potential might be a valuable indicator of protein presence in a reconstituted bilayer. Here, SDS bound to protein enhanced this potential.

On the contrary, the non-ionic detergents, such as DM or Triton X-100 have long been known to be effective in isolation and reconstitution of a number of different membrane multi-spanning proteins, such as P-type ATPases [41] or bacteriorhodopsin [42]. Triton X-100 and DM are milder detergents that lyse liposomes at more than five times higher concentration than SDS.

Triton X-100's mild character ensures proper conditions to stabilize membrane protein structures and therefore it is also used for their purification from natural membranes [43]. However, its non-ionic character and low critical micelle concentration makes it difficult to remove by dialysis. But after discovering a quick and convenient method of Triton removal by Bio-Beads [21], which was well characterized for different conditions [30], this detergent became one of the most frequently used detergents in protein reconstitution. As expected, in our experiment we were unable to remove Triton X-100 and reform liposomes even after dialyzing extensively for a period of four days. However when Bio-Beads were used, 15% reconstitution efficiency was achieved. In comparison with SDS this is a high value, though still not satisfactory. Zeta potential was lower for proteoliposomes than for control experiments without protein, although not significantly (Figure 3C). With the reconstitution efficiency far above 0% we conclude that this difference is caused by the presence of protein, since Triton X-100 is a neutral charge detergent that does not influence this potential. The resultant population of proteoliposomes was not homogeneous. Apart from the vesicles approximately 100 nm in size, a large fraction with the size of 400–500 nm was present. This implies two possibilities: we observed two populations of LUVs [36] or a larger subpopulation consisting of multilamellar vesicles (MLV). Additional data from previous studies suggests only LUVs are formed, but the authors did not observe such a large inhomogeneity [26], [30]. The characteristics of the proteoliposome population play an important role in determining their suitability for particular applications. In most cases it is critical that the liposomes are homogenous in size, size distribution and lamellarity. Therefore despite the observation of non-zero reconstitution efficiency, Triton X-100 was demonstrated as an unsuitable detergent for reconstituting apocytochrome b_6_.

The best results were obtained using dodecyl-β-D-maltoside. DM is a nonionic detergent with a low critical micellar concentration. Its hydrophobic moiety has an intermediate length, while a hydrophilic sugar headgroup is more bulky. Since the early 90's, DM has commonly been used in membrane solubilization and reconstitution of a wide range of functionally active membrane proteins [44]–[46]. Additionally DM solubilization and liposome formation from detergent-lipid micelles were examined in detail, which revealed a new “gel-like” phase in DM-lipid mixtures that strongly affects the properties of the final product [26], [47]. DM removed by dialysis yielded reconstituted vesicles with protein reconstitution efficiency of approximately 29%, however great inhomogeneity of the resultant proteoliposomes was observed (Table 3). We link this observation directly with the slow rate of detergent removal achieved in dialysis. During lysis of liposomes by DM different structures and aggregates such as planar membrane; open vesicles; long, filamentous micelles etc. arise [26]. Liposome restoration is an opposing process, therefore when DM is removed slowly, all mentioned, intermediate structures appear, which then, after ongoing fusion and mixing events, will yield inhomogeneous population at the end of the process. On the contrary, removal of DM by Bio-Beads is a fast process, in which intermediate structures do not form and the final population is homogenous in size. In our experiments, cytochrome b_6_ reconstituted with DM removed by Bio-Beads resulted in proteoliposomes with average size of around 150 nm and a narrow size distribution (Table 3). The differences in the final product properties with DM removed by dialysis and by adsorption on Bio-Beads correlates with previous observations of Lambert et.al. [26]. The reconstitution efficiency for this combination of detergent and its removal method was the highest observed and reached 46%. As for Triton X-100, for DM removed by both dialysis and Bio-Beads, a decrease in the zeta potential of the final proteoliposomes in comparison to the control was observed, and this difference proved to be statistically significant. We conclude that it reflects the highest reconstitution efficiency achieved and is in agreement with the protein net charge under the conditions used. The extramembranous fragments of protein, namely: MBP domain; N'-end; C'-end; loops between helices 1, 2 and 3 and the linker; have a net amino acid charge equal to -9.9 at the pH used in our reconstitution experiments (pH 7.4), which directly explains the negative zeta potential values, assuming the predicted reconstitution efficiency was reached. These results are in line with experiments, in which zeta potential was used to determine protein orientation in a bilayer, providing that in- and out-ends of it differs significantly in their net charge [48]. Considering these results, we advocate that zeta potential measurements can be used to quickly and conveniently test whether reconstitution was successful; having the time- and simplicity-advantage over SDS-PAGE and Western blotting or a gradient ultracentrifugation. Zeta potential measurements are applicable when outer fragments of protein are charged and nonionic detergent (for ionic detergents zeta potential might be used to control detergent removal from a membrane) and uncharged lipids are used, as these can greatly influence zeta potential of proteoliposomes [49].

Another aspect of our study was the final orientation of protein in the bilayer. The orientation of incorporated proteins might have been influenced by the method of detergent removal, as again the rate of detergent removal can be crucial. When detergent is removed quickly, the protein incorporation occurs during formation of the vesicle favoring more symmetrical orientation [50]. Slow removal of detergent leads to preferential formation of liposomes and subsequent protein incorporation, which can favor orientation asymmetry of a reconstituted protein in a bilayer [51]. These facts are valid when reconstitution starts at lytic detergent concentrations; i.e. preformed liposomes are totally lysed with a detergent and after protein addition, an isotropic mixture consisting of three components (lipid-detergent-protein) and two component (lipid-detergent) micelles forms. When sublytic detergent concentrations are used; i.e. preformed liposomes are not lysed but their bilayer is only destabilized with a detergent, the protein orientation tends to be more asymmetrical [52]. Moreover, in such a process, charged lipids can also influence the protein orientation [48].

For the reconstitution using DM removed with Bio-Beads we determined the orientation of the incorporated protein by a proteolytic assay. As shown (Figure 4), incorporation of MBP-apocytochrome b_6_ was almost ideally symmetrical (∼53% asymmetry), which confirms the established model of reconstitution method by reverse solubilization [50], [52]–[54]. On the basis of data on the reconstitution efficiency (46.5%), lipid concentration and protein content (2.5 mg/ml and 5.72 µg, respectively) in total volume (1 ml) of proteoliposomes of known size (150 nm), we calculated the number of protein molecules per one molecule of the liposome [55]. On average one liposome molecule contained 200 molecules of fusion protein incorporated into its bilayer.

From a practical point of view the reconstitution performed with Bio-Beads proved to be the most straightforward. In comparison to a dialysis it shortens the procedure from overnight to just six hours. Also handling is reduced, as Bio-Beads do not need to be changed during the reconstitution, as the dialysis buffer does; and also volumes are much lower (ca. 1 mL total volume with Bio-Beads in comparison to 100–150 mL during dialysis), which together easily allows for a parallel reconstitution of multiple samples. Also the efficiency of detergent removal (parameter not monitored in this work) is very good for Bio-Beads; reducing trace amounts of detergent and improving liposome stability and impermeability [50].

## Conclusions

In this work, using apocytochrome b_6_, we systematically compared its reconstitution into liposomes using three different detergents: SDS, Triton X-100 and DM, and two methods of their removal: slow – by dialysis and fast – using Bio-Beads. Despite the fact that two of the detergents used (Triton X-100 and DM) proved to successfully renature the protein to its native, helical rich structure after protein purification, their application in a reconstitution was dramatically different. Only DM removed with Bio-Beads gave satisfactory reconstitution efficiency – a crucial factor if downstream applications are considered such as activity measurements – and yielded proteoliposome populations of homogenous size and distribution. This points to the fact, that when protein reconstitution is considered, a detergent is the most crucial factor responsible for the end product parameters. Therefore, detergent-free methods of protein purification (e.g. from heterologous expression systems and not from native membranes) are advantageous here, as they do not predetermine the choice of a detergent to be used in a reconstitution.

Additionally we showed that zeta potential could be used as a convenient parameter for a quick assessment of reconstitution success and having an indisputable advantage in time and simplicity over e.g. SDS-PAGE followed by Western blotting or gradient ultracentrifugation. Moreover the same parameter allows monitoring the removal of ionic detergents, if such were used.
